# Impact of FXIII Levels on Hematoma Expansion and Outcomes in Patients with Spontaneous Intracranial Hemorrhage: A Prospective Observational Cohort Study of 132 Patients

**DOI:** 10.1007/s12028-026-02513-3

**Published:** 2026-04-03

**Authors:** Li Wang, Yahui Luo, Qiang Yuan, Xuehua Xiong, Guanpeng Jin, Jiangbo Dong, Jianfeng Zhou, Ke Lv

**Affiliations:** 1 The Center for Clinical Molecular Medical Detection, Innovative and Translational Laboratory of Molecular Diagnostics, Laboratory Medicine Center, The First Affiliated Hospital of Chongqing Medical University, No.1 Youyi Road, Chongqing, 400016 China; 2 Department of Neurosurgery, Laboratory of Neurological Diseases and Interdisciplinary Medicine, The First Affiliated Hospital of Chongqing Medical University, No.1 Youyi Road, Chongqing, 400016 China; 3 Department of Neurosurgery, Dazhou Dachuan District People’s Hospital, Dazhou Third People’s Hospital), No.700 Hanxing North Street, Sanliping Street, Dachuan District, Dazhou, 635000 China; 4 Chongqing Emergency Medical Center, Chongqing University Central Hospital, School of Medicine, Chongqing University, Chongqing, 400014 China; 5 Department of Neurosurgery, Huashan Hospital, Fudan University, No.12 Wulumuqi Middle Road, Shanghai, 200040 China

**Keywords:** Spontaneous intracerebral hemorrhage, Coagulation factor XIII, Hematoma expansion, Mortality, Functional outcome

## Abstract

**Background:**

Diminished levels of FXIII are significantly correlated with hematoma expansion (HE) and unfavorable clinical outcomes. Although the crucial function of FXIII in coagulation is well-recognized, the connection between FXIII deficiency and either HE or negative prognosis in patients with spontaneous intracerebral hemorrhage (sICH) is not well understood.

**Methods:**

Patients with sICH were confirmed through baseline computed tomography scans conducted within 6 h of symptom onset. Plasma FXIII levels were assessed at the time of admission using a standardized enzyme-linked immunosorbent assay. Restricted cubic spline models were employed to explore potential nonlinear relationships between admission FXIII levels and both hematoma expansion and clinical outcomes. Multivariable logistic regression analyses were performed to ascertain the association between FXIII levels and functional outcomes at 3 months postonset.

**Results:**

In the study cohort of 132 patients with sICH, 49 experienced HE. The median admission level of FXIII was significantly lower in patients with HE, with a median [IQR] of 8.21 mg/L (6.33, 10.37), compared with those without HE, who had a median value [IQR] of 11.68 mg/L (8.49, 21.20), *p* < 0.001. A deficiency in Factor XIII, defined as a plasma level < 10 mg/L, was associated with HE, with a fivefold increase in risk as indicated by an adjusted odds ratio of 5 (95% CI 2.031–12.325; *p* < 0.001). Furthermore, multivariable analysis revealed that this deficiency was associated with poor functional outcomes, defined as a modified Rankin Scale (mRS) score of > 3, by a factor of 59.45 (OR 59.45, 95% CI 7.258–487.006; *p* < 0.001), and increased the risk of mortality by a factor of 12.07 (OR 12.07, 95% CI 2.526–57.669; *p* = 0.002) in patients with sICH.

**Conclusions:**

In a prospective observational cohort study of patients with sICH, FXIII deficiency was found to be associated with HE and poor prognosis.

**Supplementary Information:**

The online version contains supplementary material available at https://doi.org/10.1007/s12028-026-02513-3.

## Background

Over 3.3 million people globally died from spontaneous intracerebral hemorrhage (sICH) in 2021. sICH is acknowledged worldwide as the deadliest type of stroke and accounts for about half of the disease burden attributed to strokes, making it a significant global public health issue [1]. The pathophysiology of sICH involves complex processes such as mechanical mass effect, hematoma expansion, and secondary injury, with preventing hematoma expansion (HE) being a key therapeutic goal. [2]. Coagulation factor XIII (FXIII) is crucial for hemostasis as it stabilizes and strengthens the structure of fibrin clots. By facilitating intermolecular fibrin cross-linking, FXIII converts loose fibrin aggregates into robust hemostatic barriers [3]. FXIII deficiency weakens the stability of clots, causing thrombi to be vulnerable to fragmentation and breakdown, which contributes to the expansion of intracranial hematomas and aggravates secondary brain damage [4]. Moreover, a lack of FXIII leads to poor wound healing, ongoing subcutaneous bleeding tendencies, and potentially fatal spontaneous hemorrhages in the central nervous system (CNS) [5–7].

Lower FXIII levels are significantly linked to a higher risk of intracranial hematoma expansion. In a study involving 32 patients with acquired FXIII autoantibodies, about 13% experienced intracranial hematomas linked to decreased FXIII levels [8]. Studies indicate that in postcraniotomy patients, postoperative FXIII activity below 60% independently predicts intracranial hemorrhage, increasing the risk of hematoma expansion by 6.4 times [9]. The study by Gerlach et al. also confirmed that intracranial surgery patients with diminished FXIII levels face a greater likelihood of experiencing postoperative intracranial hemorrhage [10]. Moreover, even slightly lowered FXIII levels greatly increase the risk of bleeding, such as intracranial hemorrhage, especially when combined with dilutional coagulopathy or during trauma or surgery [11]. A different study emphasized the combined effect of fibrinogen and FXIII, showing that reducing both factors together significantly raise the risk of intracranial hemorrhage after surgery [12]. According to the 5th and 6th editions of the European Guideline on managing major bleeding and coagulopathy following trauma, FXIII testing is recommended for trauma patients, and FXIII substitution therapy is suggested for those with low levels [13]. Currently, the practices of screening and supplementing for FXIII deficiency are primarily restricted to patients with congenital FXIII deficiency and some instances of severe traumatic brain injury. Despite the increasing awareness of FXIII’s vital role in coagulation, there remains insufficient evidence about its connection to outcomes in patients with sICH.

In this prospective observational cohort study involving patients with spontaneous intracerebral hemorrhage, we evaluated the influence of admission FXIII levels on patient outcomes, specifically focusing on hematoma expansion and neurological function. The outcomes of this research are anticipated to inform clinical practice by providing an evidence base for FXIII screening and supplementation in patients with sICH.

## Materials and Methods

### Patient Population

This is a prospective observational cohort study involving patients with sICH. The patients with sICH presenting to the Neurological Stroke Center between September 2022 and April 2025 were enrolled. The primary end point assessed the association between admission FXIII levels and prognosis in patients with sICH. Inclusion criteria comprised: patients were aged > 18, and presented within 6 h of onset; Patients had symptomatically and radiologically confirmed sICH. Patients with traumatic ICH, hemorrhagic transformation of ischemic stroke, history of chronic bleeding disorders, cerebrovascular malformations, intracranial aneurysms, or cerebral amyloid angiopathy (CAA) were excluded. Additionally, patients with neurosurgical interventions (hemicraniectomy and/or clot evacuation) between baseline and follow-up head computed tomography (CT) scans, preexisting coagulopathy, concurrent use of anticoagulant or antiplatelet agents, and preexisting severe hepatic insufficiency were excluded to minimize relevant confounders. The protocol for this study has been approved by the ethics committee of the First Affiliated Hospital of Chongqing Medical University.

### Data Collection

The data collected from patients with sICH encompassed demographic variables (age, sex, history of hypertension) and clinical variables including systolic blood pressure (SBP), mean arterial pressure (MAP), Glasgow Coma Scale (GCS) score, spot sign and Original Intracerebral Hemorrhage (OICH) scale score [14, 15] on admission. Within 6 h following intracerebral hemorrhage (ICH), coagulation parameters—specifically the international normalized ratio (INR), prothrombin time (PT), activated partial thromboplastin time (APTT), fibrinogen (FIB), and D-dimer—were analyzed at a Central Clinical Chemistry Laboratory using standardized assays. Concurrently, routine hematological and biochemical parameters, including red blood cell count (RBC), white blood cell count (WBC), hemoglobin (Hb), platelet count, sodium (Na), glucose, and creatinine levels, were measured and documented as part of the standardized laboratory assessment. Plasma FXIII levels in patients with sICH were measured at a central laboratory on admission using the Human Factor XIII ELISA Kit (Assaypro, France).

All patients underwent noncontrast CT scans at baseline and 24 h later; CT angiography was performed immediately after the baseline scan for all patients. All patients underwent rigorous blood pressure management aimed at maintaining systolic blood pressure below 140 mmHg, in accordance with current guidelines for the management of intracerebral hemorrhage. The primary outcome was hematoma expansion on follow-up CT, defined as an absolute increase in intracerebral hemorrhage volume of > 6 mL or a relative increase of > 33% compared with baseline CT. Hematoma volume, calculation of the hematoma volume can be easily performed with the ABC/2 method (cm^3^). The OICH score was used to assess clinical grading in patients with sICH. The 90-day Modified Rankin Scale (mRS) was assessed through clinic visits or via telephone interviews. mRS score of 0–2 was considered indicative of a good functional outcome [16]. The “spot sign” refers to a region of contrast enhancement observed within a primary intracerebral hemorrhage on computed tomographic (CT) angiogram source images. Surgical interventions are defined as hematoma evacuation, external ventricular drainage, or decompressive craniectomy. We categorized the location of sICH into three groups: deep gray matter, infratentorial, and lobar [17, 18].

### Statistical Analyses

Continuous data are reported as mean ± SD or median (IQR); categorical data as percentages. Group comparisons used chi-square tests (categorical), parametric tests (*t*-test/analysis of variance (ANOVA) for normal data), or nonparametric tests (Mann–Whitney *U*/Kruskal–Wallis for non-normal data), following homogeneity assessment (Levene’s test). FXIII-outcome relationships were modeled with linear and cubic splines. Backward stepwise logistic regression identified predictors of hematoma expansion and 3-month mortality/functional outcomes. Analyses used R v3.3.6 and SPSS v25.0. Statistical significance was defined as two-sided *p* < 0.05.

## Results

**Fig. 1:**
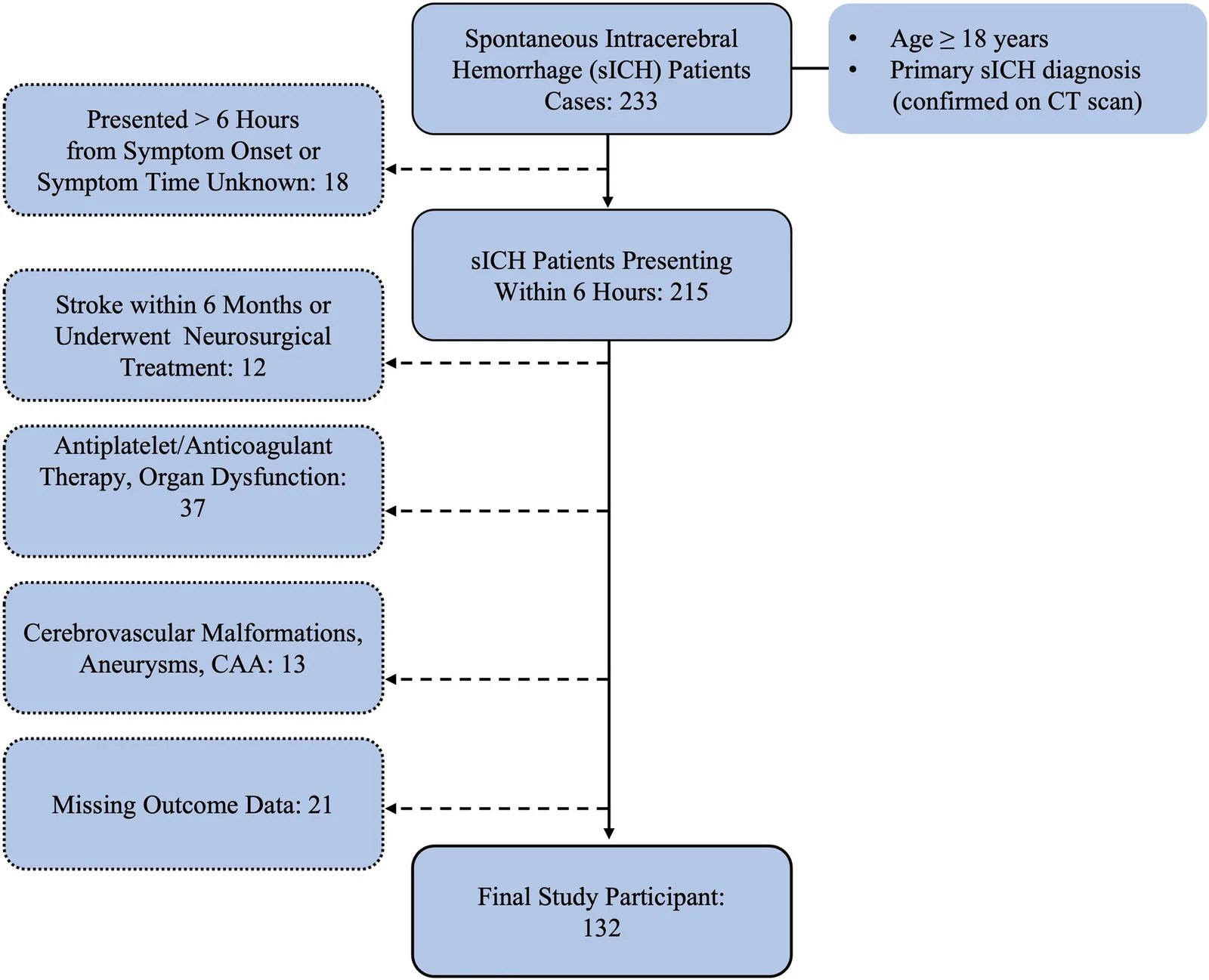
Study flowchart. CT, computed tomography; sICH, spontaneous intracerebral hemorrhage; CAA, cerebral amyloid angiopathy

The inclusion and exclusion criteria for this study are detailed in Fig. 1. A cohort of 132 patients diagnosed with spontaneous intracerebral hemorrhage (sICH) was recruited for this investigation. The median age of the participants was 59 years, with an interquartile range (IQR) of 51–70.25 years, and the sample comprised 92 males, representing 69.7% of the total. A significant proportion of the patients with sICH, specifically 93 individuals (70.45%), had a history of hypertension. The mean systolic blood pressure (SBP) and mean arterial pressure (MAP) recorded for the cohort were elevated, measuring 165.9 ± 26.63 mmHg and 120.43 ± 19.41 mmHg, respectively. Upon admission, the Glasgow Coma Scale (GCS) scores varied from 3 to 15, with a median score of 11 and an IQR of 8–13. The Original Intracerebral Hemorrhage (OICH) score at admission had a median value of 2, with an IQR of 1–3. The baseline hematoma volume was characterized by a median of 15.89 ml and an IQR of 8–39.5 ml. The majority of hematomas were located in the deep gray matter (87 cases, 66.41%), followed by lobar (27 cases, 20.61%) and infratentorial (17 cases, 12.98%) regions, with 61 patients (46.21%) presenting with intraventricular hemorrhage. Enhanced CT scans indicated that 50% of the hematomas (66 cases) exhibited the spot sign. Upon admission, the FXIII levels in the majority of patients were significantly lower than the reference threshold of 20 mg/L, with a median [IQR] of 9.81 [7.88, 18.1] mg/L (Table 1).

**Table 1:** Baseline characteristics of patients with sICH with vs without hematoma expansion (HE)

	Total (*n* = 132)	None HE (*n* = 93)	HE (*n* = 39)	*p* Value^*a*^
Age, Median [IQR]	59 (51, 70.25)	58 (51, 68)	61 (55, 76.5)	0.075
Gender, *n* (%)				0.126
Male	92 (69.7)	69 (74.19)	23 (58.97)	
Female	40 (30.3)	24 (25.81)	16 (41.03)	
Hypertension, *n* (%)				0.096
No	39 (29.55)	23 (24.73)	16 (41.03)	
Yes	93 (70.45)	70 (75.27)	23 (58.97)	
SBP, Mean ± SD	165.9 ± 26.63	165.59 ± 27.86	166.64 ± 23.76	0.837
MAP, Mean ± SD	120.43 ± 19.41	121.42 ± 20.54	118.08 ± 16.39	0.368
OICH, Median [IQR]	2 (1, 3)	2 (1, 3)	2 (1, 3)	0.223
GCS at admission, Median [IQR]	11 (8, 13)	11 (8, 13)	10 (7.5, 12)	0.074
Na (mmol/L), Median [IQR]	139.3 (137.3, 142)	139.3 (137.5, 142)	139.3 (136.2, 142)	0.686
Glucose (mmol/L), Median [IQR]	7.95 (6.8, 9.77)	8 (6.9, 9.7)	7.8 (6.5, 10.2)	0.675
RBC (× 10^12^/L), Median [IQR]	4.54 (4.02, 4.96)	4.75 (4.08, 5.03)	4.38 (3.98, 4.84)	0.13
Creatinine (µmol/L), Median [IQR]	66.35 (54.98, 79.78)	66 (54.4, 79.1)	68 (58, 83.4)	0.662
INR, Median [IQR]	0.99 (0.95, 1.04)	0.99 (0.95, 1.02)	0.99 (0.95, 1.06)	0.505
APTT(s), Median [IQR]	31.15 (26.87, 33.9)	31.5 (28, 33.9)	29.8 (25.45, 32.25)	0.151
WBC (× 10^9^/L), Median [IQR]	10.28 (8, 12.94)	10.22 (7.94, 13.12)	10.51 (8.43, 12.25)	0.663
PT(s), Median [IQR]	12.8 (11.9, 13.22)	12.8 (12.1, 13.3)	12.8 (11.65, 13.2)	0.768
FIB (g/L), Median [IQR]	3.04 (2.54, 3.52)	3.09 (2.55, 3.55)	2.88 (2.45, 3.39)	0.287
D-dimer (mg/L), Median [IQR]	0.56 (0.35, 1.09)	0.51 (0.32, 1.03)	0.61 (0.45, 1.54)	0.079
Platelet (× 10^9^/L), Median [IQR]	197 (160, 227.25)	201 (161, 235)	189 (160, 219)	0.202
Hemoglobin (g/L), Median [IQR]	138 (123.75, 148)	139 (127, 149)	136 (123, 146)	0.375
Baseline Volume (ml), Median [IQR]	15.89 (8, 39.5)	15.79 (8, 45)	21 (10.93, 32.53)	0.521
Location, *n* (%)				0.49
Deep gray matter	88 (66.67)	61 (65.60)	27 (69.23)	
Infratentorial	17 (12.88)	14 (15.05)	3 (7.69)	
Lobar	27 (20.45)	18 (19.35)	9 (23.08)	
Presence of IVH, *n* (%)				1
No	71 (53.79)	50 (53.76)	21 (53.85)	
Yes	61 (46.21)	43 (46.24)	18 (46.15)	
Spot sign, *n* (%)				< 0.001
No	66 (50)	56 (60.22)	10 (25.64)	
Yes	66 (50)	37 (39.78)	29 (74.36)	
FXIII (mg/L), Median [IQR]	9.81 (7.88, 18.1)	11.68 (8.49, 21.2)	8.21 (6.33, 10.37)	< 0.001
FXIII (mg/L), *n* (%)				0.002
> 10 mg/L	63 (47.73)	53 (56.99)	10 (25.64)	
< 10 mg/L	69 (52.27)	40 (43.01)	29 (74.36)	
mRS, Median [IQR]	4 (2, 5)	4 (2, 5)	5 (4, 5)	0.01
mRS, *n* (%)				0.012
mRS 0–2	35 (26.52)	31 (33.33)	4 (10.26)	
mRS 3–6	97 (73.48)	62 (66.67)	35 (89.74)	

sICH, spontaneous intracerebral hemorrhage; HE, hematoma expansion; SBP, systolic blood pressure; MAP, mean arterial pressure; GCS, Glasgow Coma Scale; OICH, original intracerebral hemorrhage scale; RBC, red blood cell; WBC, white blood cell; INR, international normalized ratio; PT, prothrombin; APTT, activated partial thromboplastin time; FIB, fibrinogen; FXIII, coagulation factor XIII; mRS, modified Rankin Scale

^*a*^* P* values not adjusted for multiple comparisons. Statistical significance determined by chi-square test, Student’s *t*-test or Mann–Whitney *U* tests

In this study, we initially conducted a univariate analysis to compare baseline characteristics and variables between patients with and without HE. Following this, a backward stepwise logistic regression was utilized. Variables that exhibited a significance level of *p* < 0.1 in the univariate analysis were included to identify those independently associated with HE. As a result, age, hypertension, GCS score, D-dimer levels, the presence of a Spot Sign, and FXIII levels were included in the multivariable analysis. Hematoma volume was excluded from the multivariable model due to its lack of significant difference in the univariate analysis.

### Hematoma Expansion

**Fig. 2:**
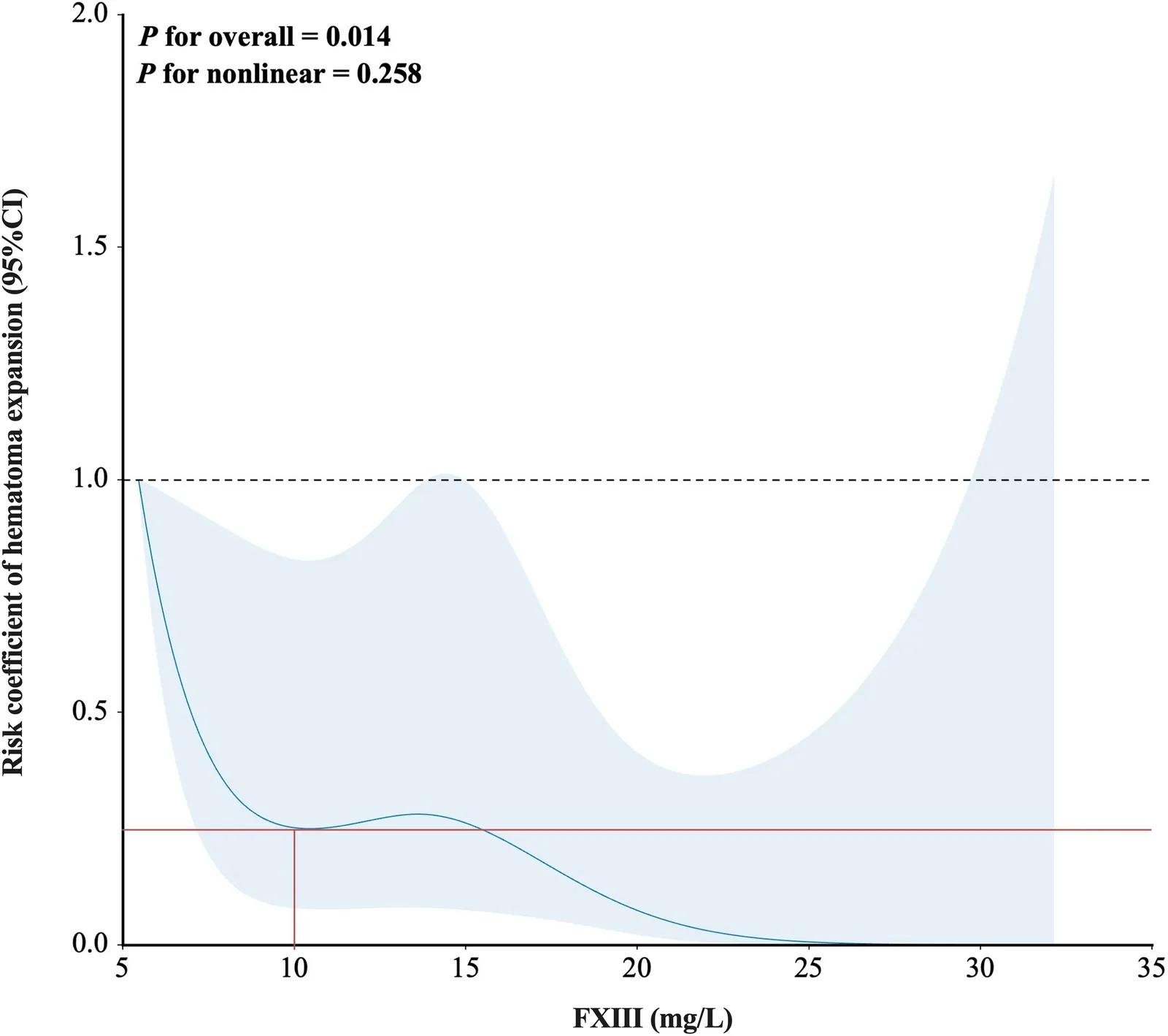
The shape of the relationship between the FXIII levels at admission and hematoma expansion (HE) risk. When FXIII levels < 10 mg/L, the risk of HE increases progressively with declining FXIII levels

Hematoma expansion (HE) was observed in 39 (29.5%) of the 132 patients enrolled with sICH. Patients with HE exhibited significantly lower admission levels of FXIII, with a median [IQR] of 8.21 [6.33, 10.37] mg/L, compared with non-HE patients with sICH, who had a median [IQR] of 11.68 [8.49–21.20] mg/L (*p* < 0.001). Among the 39 patients with HE, 29 (76%) tested positive for the spot sign (*p* < 0.001) (Table 1). Nonlinear analysis revealed an inverse correlation between admission FXIII levels and the risk of HE, with this trend being most pronounced at FXIII levels of 10 mg/L (Fig. 2). Analysis of continuous variables indicated that both FXIII levels (OR = 0.83; 95% CI, 0.76–0.914; *p* < 0.001) and the presence of the spot sign (OR = 6.16; 95% CI, 2.465–15.373; *p* < 0.001) were significantly associated with HE. Multivariable logistic regression analysis, with FXIII dichotomized at 10 mg/L, further highlighted this trend: patients with FXIII levels below 10 mg/L had a fivefold increased likelihood of experiencing HE compared with those with levels above 10 mg/L (OR = 5; 95% CI, 2.031–12.325; *p* < 0.001) (Table 2).

**Table 2:** Correlation between FXIII levels and hematoma expansion (HE)

Variables	OR (95% CI)	*p *value
HE with continuous FXIII		
FXIII level	0.83(0.76–0.914)	< 0.001
Spot sign	6.16(2.465–15.373)	< 0.001
HE with binary FXIII		
FXIII level < 10 mg/L	5(2.031–12.325)	< 0.001
Spot sign	5.61(2.286–13.768)	< 0.001

### Mortality

**Fig. 3:**
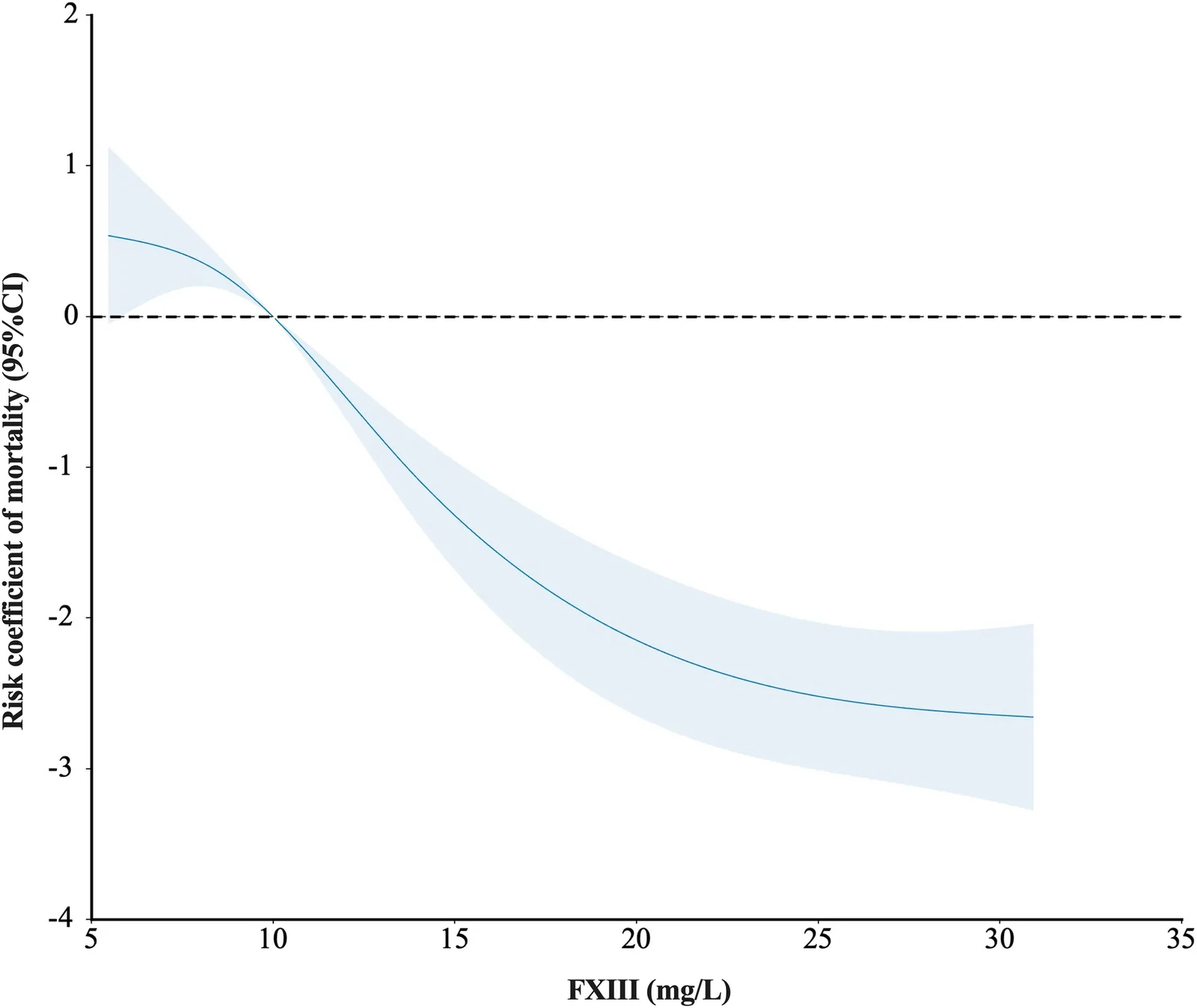
The shape of the relationship between the FXIII levels and mortality. When FXIII levels falls 10 mg/L, patient mortality increases demonstrably with further decline in FXIII levels

The admission GCS scores demonstrated a statistically significant difference between survivors and nonsurvivors, with median [IQR] of 11 [9, 13] and 7 [4, 10], respectively (*p* < 0.001). Similarly, the OICH scores exhibited a pronounced disparity, with survivors having median [IQR] of 1 [1, 2] compared to 3 [2, 4] in nonsurvivors (*p* < 0.001). Admission glucose levels also showed significant differences between the groups, with survivors having median levels [IQR] of 7.6 [6.55, 9.1] mmol/L, whereas nonsurvivors had 9.3 [8, 12.1] mmol/L (*p* < 0.001). Furthermore, baseline hematoma volumes were significantly lower in survivors, with a median [IQR] of 14.22 [8–31.5] ml, compared with 49 [13.54–74] ml in nonsurvivors (*p* < 0.001). FXIII levels were notably higher in survivors, with a median [IQR] of 11.68 [8.54–19.15] mg/L, as opposed to 7.13 [5.95–8.34] mg/L in nonsurvivors (*p* < 0.001). Further analysis indicated that among deceased patients, over 93% had FXIII levels < 10 mg/L (*n* = 27, 93.1%; *p* < 0.001), whereas only approximately 40% of surviving patients exhibited FXIII levels < 10 mg/L (*n* = 42, 40.78%; *p* < 0.001) (Supplementary Table 1). The investigation into the nonlinear association between FXIII levels and patient mortality demonstrated that FXIII concentrations below the 10 mg/L threshold were linked to a marked increase in mortality rates (Fig. 3). In multivariate logistic regression analyses treating FXIII as a continuous variable, a decline in FXIII levels was significantly correlated with heightened patient mortality (OR = 0.84; 95% CI, 0.737–0.959; *p* = 0.009). Additionally, both the Glasgow Coma Scale (GCS) score and glucose levels were found to be significantly associated with mortality, with odds ratios of 0.79 (95% CI, 0.679–0.929; *p* = 0.004) and 1.28 (95% CI, 1.051–1.568; *p* = 0.014), respectively. Furthermore, when FXIII levels were dichotomized at the 10 mg/L threshold in multivariable logistic regression models, individuals with FXIII levels < 10 mg/L exhibited a 12.07-fold increased risk of mortality (OR = 12.07; 95% CI, 2.526–57.669; *p* = 0.002). The associations of GCS score and glucose levels with mortality remained significant across both patient cohorts stratified by the FXIII cutoff of 10 mg/L, with odds ratios of 0.8 (95% CI, 0.685–0.944; *p* = 0.008) and 1.27 (95% CI, 1.05–1.542; *p* = 0.014), respectively (Table 3).

**Table 3:** Correlation between FXIII levels and mortality

Variables	OR (95% CI)	*p *value
Mortality with continuous FXIII		
Glasgow Coma Scale	0.79(0.679–0.929)	0.004
Glucose	1.28(1.051–1.568)	0.014
FXIII level	0.84(0.737–0.959)	0.009
Mortality with binary FXIII		
Glasgow Coma Scale	0.8(0.685–0.944)	0.008
Glucose	1.27(1.05–1.542)	0.014
FXIII level < 10 mg/L	12.07(2.526–57.669)	0.002

### 90-Day Functional Outcome

**Fig. 4:**
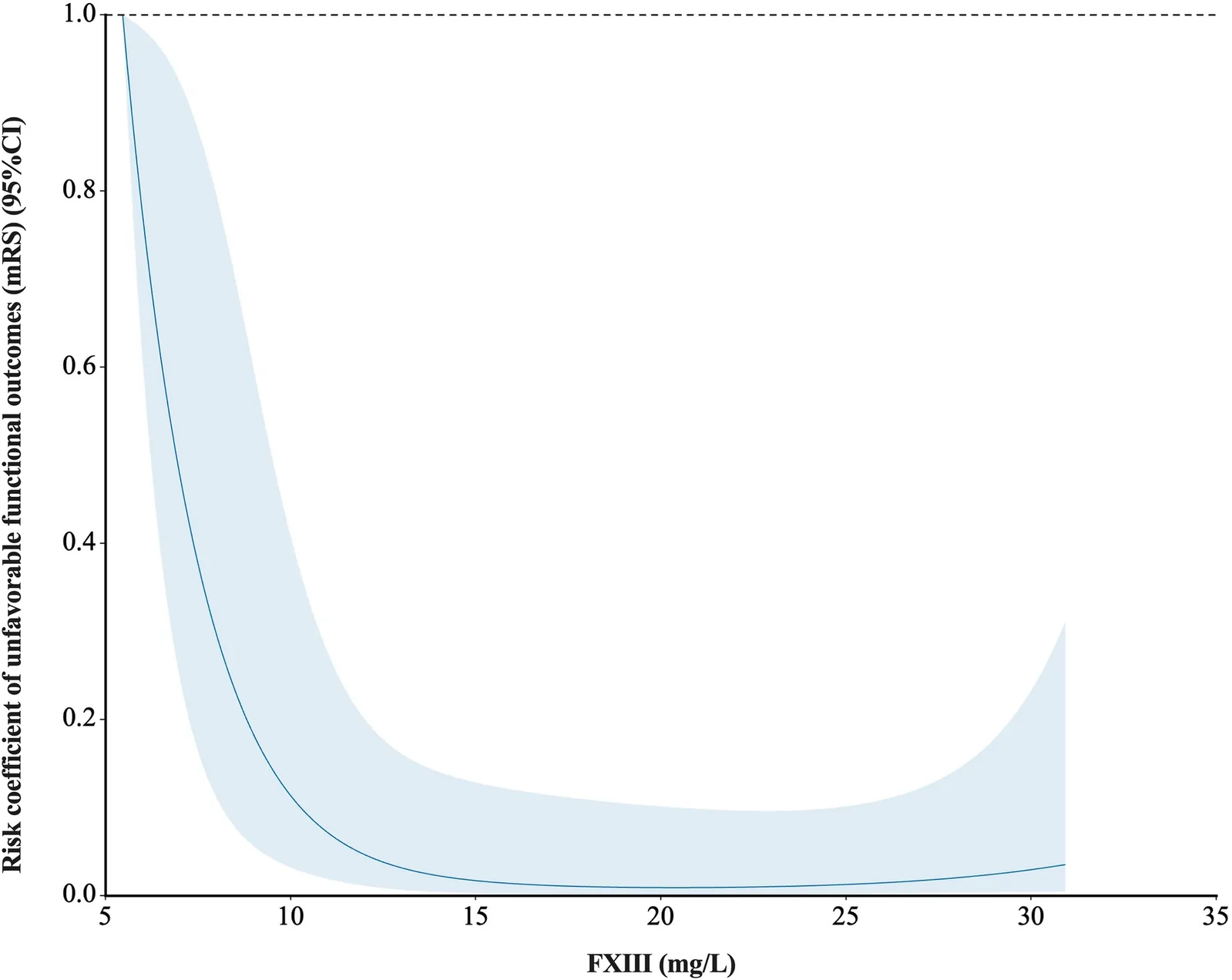
The shape of the relationship between the FXIII levels and unfavorable functional outcome (mRS) prediction. Elevated FXIII levels correlate with reduced mRS scores and increased probability of favorable functional outcomes. mRS, modified Rankin Scale

In this study, 73.48% of patients exhibited unfavorable functional outcomes, defined as a modified Rankin Scale (mRS) score of 3 or higher (*n* = 97). Patients who achieved favorable outcomes (mRS < 2) presented with significantly higher Glasgow Coma Scale (GCS) scores upon admission compared with those with poor outcomes, with a median [IQR] of 14 [12.5–14.5] versus 10 [7–12], respectively (*p* < 0.001). Similarly, the OICH score exhibited a consistent pattern, with patients experiencing unfavorable outcomes having higher OICH scores than those with favorable outcomes, reflected by a median OICH [IQR] of 2 [1–3] versus 1 [1, 2] (*p* < 0.001). In examining the influence of FXIII levels on patient prognosis, the group with favorable outcomes demonstrated a significantly higher median FXIII [IQR] of 22.18 [18.08, 26.69] mg/L, compared with 9.00 [7.13, 11.09] mg/L in the group with unfavorable outcomes (*p* < 0.001). This positive correlation persisted when patients were stratified by predefined FXIII thresholds: among the 97 patients with unfavorable functional outcomes, over 70% had FXIII levels < 10 mg/L, whereas only 2.86% of the favorable outcomes group exhibited FXIII levels < 10 mg/L (*p* < 0.001) (Supplementary Table 2). Furthermore, analysis of the nonlinear relationship between admission FXIII levels and mRS indicated that as FXIII levels increased, there was a corresponding decline in mRS scores (Fig. 4). The analysis of continuous variables identified FXIII levels (OR = 0.74; 95% CI, 0.66–0.839; *p* < 0.001), D-dimer (OR = 2.43; 95% CI, 1.342–4.385; *p* = 0.003), and GCS (OR = 0.5; 95% CI, 0.352–0.707; *p* < 0.001) as factors significantly associated with patient functional outcomes. This relationship was further intensified in the multivariable logistic regression analysis for the mRS when FXIII was dichotomized at 10 mg/L. Specifically, patients with FXIII levels below 10 mg/L demonstrated a 59.45-fold increased risk of poor functional outcomes (mRS > 3) compared with those with FXIII levels above 10 mg/L (OR = 59.45; 95% CI, 7.258–487.006; *p* < 0.001) (Table 4).

**Table 4:** Correlation between FXIII levels and functional outcome (mRS)

Variables	OR (95% CI)	*p *value
Functional outcome with continuous FXIII		
Glasgow Coma Scale	0.5(0.352–0.707)	< 0.001
D-dimer	2.43(1.342–4.385)	0.003
FXIII level	0.74(0.66–0.839)	< 0.001
Functional outcome with binary FXIII		
Glasgow Coma Scale	0.59(0.443–0.777)	< 0.001
FXIII level < 10 mg/L	59.45(7.258–487.006)	< 0.001

## Discussion

A deficiency in FXIII is strongly associated with visceral hemorrhage, delayed postoperative bleeding, and intracranial hemorrhage [19–21]. This study provides new evidence demonstrating that admission levels of FXIII are independently correlated with hematoma expansion, mortality, and functional outcomes in patients with sICH. Our analysis of factors associated with HE indicates that plasma FXIII levels of < 10 mg/L are inversely associated with the risk of HE, thereby establishing a clinically relevant threshold for evaluating the risk of poor prognosis. Furthermore, we investigated the relationship between FXIII levels and clinical outcomes, including mortality and functional status. Patients with FXIII levels < 10 mg/L demonstrated significantly higher mortality rates and a greater incidence of unfavorable functional outcomes compared with those with levels > 10 mg/L. As the final catalyst in the coagulation cascade, FXIII is crucial for stabilizing fibrin clots through γ-chain crosslinking. A deficiency in FXIII may compromise clot stability, leading to accelerated fibrinolysis, which could account for its association with hematoma expansion and adverse clinical outcomes [22]. An additional significant contribution of this research is the identification of a potential necessity for accelerated repeat noncontrast head CT monitoring in patients with sICH who present with FXIII levels of < 10 mg/L.

A comprehensive retrospective cohort study identified that a subset of patients with traumatic brain injury (TBI) exhibited intracranial hematoma expansion despite having elevated fibrinogen levels above the normal reference range [23]. This unexpected observation highlights the need to explore the collaborative roles of other coagulation factors in addition to fibrinogen. In response, we conducted an interaction analysis to assess the combined effects of fibrinogen and FXIII on hematoma progression. The cohort has maintained fibrinogen levels within normal limits (median [IOR]: 3.04 g/L [2.54, 3.52]). Importantly, while FXIII levels were significantly reduced in patients experiencing HE, fibrinogen levels did not show a statistically significant decrease in groups with HE, mortality, or poor functional outcomes. These results emphasize the importance of monitoring FXIII levels, even when fibrinogen levels are within normal ranges.

In the spontaneous intracerebral hemorrhage cohort, coagulation profiling indicated that the international normalized ratio (INR), prothrombin time (PT), and activated partial thromboplastin time (APTT) were not prolonged across various subgroups. Hematologic parameters, including red blood cell count (RBC), white blood cell count (WBC), platelet count, and hemoglobin levels, remained stable. An isolated increase in D-dimer levels has been independently correlated with negative functional outcomes. Although traditional plasma-based coagulation assays, including PT and APTT, are proficient in detecting coagulopathy in patients undergoing anticoagulation therapy, their effectiveness is limited in identifying coagulation abnormalities in nonanticoagulated individuals and in forecasting the risk of intracranial hematoma expansion. This limitation is attributable to their original design, which was not intended to evaluate coagulopathy during active hemorrhagic events [23, 24]. These findings highlight the critical importance of conducting admission FXIII testing to predict hematoma expansion and clinical outcomes in patients with sICH, especially among those with normal routine coagulation profiles. No significant differences were detected in admission sodium or creatinine levels. Importantly, nonsurvivors demonstrated significantly elevated admission glucose levels (median [IQR]: 9.3 mmol/L [8, 12.1]) compared with survivors (median [IOR]: 7.6 mmol/L [6.55, 9.1]). Hyperglycemia during the acute phase of sICH, regardless of diabetes status, consistently predicts poor prognosis. Elevated glucose levels exacerbate cerebral tissue damage, promote edema formation, expand the ischemic penumbra, and increase both mortality and disability, while also delaying recovery [25, 26]. Therefore, rigorous glycemic control is a crucial therapeutic strategy for enhancing outcomes in patients with sICH.

ICH constitutes the second most prevalent stroke subtype, with a higher incidence observed among Asian populations, older adults, and males [27]. Our study findings underscore that the elder adults continue to represent the primary demographic affected by spontaneous hypertensive intracerebral hemorrhage, with a significantly higher incidence rate observed in male patients compared with female patients. Notably, despite some patients reporting no prior history of hypertension, they exhibited markedly elevated blood pressure levels upon admission, with a mean systolic blood pressure (SBP) of 165.9 ± 26.63 mmHg and a mean arterial pressure (MAP) of 120.43 ± 19.41 mmHg. Prompt and intensive blood pressure management is crucial, as it reduces the risk of hematoma expansion and neurological decline [28, 29]. In accordance with clinical guidelines, all patients enrolled with spontaneous hypertensive intracerebral hemorrhage had their systolic blood pressure reduced to below 140 mmHg (ideally 130 mmHg) following the onset of ICH. Antihypertensive therapy was initiated within 1 h of hospital admission for all cases.

The OICH Score has been validated as a fundamental prognostic indicator for patients through numerous clinical studies [14, 15]. Although our research revealed its limitations in predicting intracranial hematoma expansion, the OICH Score retained its efficacy in forecasting adverse functional outcomes and mortality. Current evidence advocates for the integration of OICH and GCS scores in critical triage decisions, specifically to identify patients with sICH at elevated risk of poor prognosis who require treatment at comprehensive stroke centers [30]. Hematoma volume serves as a crucial prognostic factor, influencing outcomes through multifaceted mechanisms, including direct neural injury, intracranial hypertension, and secondary pathological cascades [31–33].

A primary limitation of this study is the reliance on measuring FXIII levels instead of assessing its functional activity. In the acute clinical context of spontaneous intracerebral hemorrhage, evaluating antigen levels provided a practical and preliminary assessment of FXIII status. Nonetheless, the absence of functional activity assays precludes a comprehensive characterization of the coagulation disorder associated with reduced FXIII levels. Moreover, the lack of differentiation between specific time points within the initial 6 h may not have adequately accounted for the potential impact of onset-to-scan time on hematoma expansion. Another limitation of this study is its concentrated focus on FXIII, which consequently resulted in an insufficient assessment of other factors, such as factor VII and hereditary FXIII deficiency. This enabled a specific analysis of FXIII’s role but limited our ability to fully assess the comprehensive coagulation dysfunction in sICH. The stringent inclusion criteria of the clinical trial may limit the generalizability of the findings. The single-center, prospective registry-based retrospective design could introduce selection bias; thus, future multicenter clinical studies are recommended to enhance the reliability of the results. The geographical homogeneity of the study population may also limit the applicability of the findings to diverse populations. The relatively small sample size (*n* = 132) reduced the statistical power for conducting subgroup analyses of FXIII deficiency subtypes. The absence of longitudinal FXIII monitoring precluded causal inference regarding FXIII consumption versus compensatory synthesis during hematoma evolution. An additional limitation of our study is the absence of functional global hemostatic assays, such as thromboelastography (TEG) or rotational thromboelastometry (ROTEM), in our evaluation. Although we identified a correlation between reduced FXIII levels and hematoma expansion, these point-of-care viscoelastic tests would have offered a more comprehensive assessment of the patients’ overall coagulation status. The lack of such data precludes us from definitively characterizing the complete spectrum of coagulopathy associated with low FXIII levels or from excluding the presence of concurrent coagulation abnormalities. Future research should incorporate both specific factor level measurements and functional viscoelastic testing to elucidate the coagulation dynamics in cases of sICH [34]. Another limitation of this study was the assessment of hematoma volume using the ABC/2 method. Although this approach is clinically practical, it demonstrates lower accuracy compared with manual or computer-assisted volumetric analyses, thereby representing a methodological constraint [35]. This study did not explore the precise etiology of reduced FXIII levels, such as whether they are consumptive or autoimmune in origin, nor did it assess the presence of anti-FXIII autoantibodies. Consequently, this limitation prevents the formulation of definitive conclusions concerning the underlying mechanism of the deficiency. Although patients who received blood products prior to admission were excluded, the potential dilution effects from preadmission fluid administration volumes were not considered, which may have influenced baseline FXIII levels. Furthermore, although blood samples were collected within 6 h following the ictus, the variability in sampling times might have impacted the FXIII measurements. A further potential limitation of this study is the heightened risk of a Type I error resulting from the numerical imbalance between groups, such as that observed between HE-positive cases and HE-negative controls. To bolster the robustness of our findings, we primarily base our conclusions on multivariable-adjusted analyses conducted through logistic regression. This analytical approach partially addresses the structural imbalance while simultaneously controlling for key confounding variables.

## Conclusions

Our findings indicate that reduced FXIII levels upon admission are associated with an increased risk of HE and adverse clinical outcomes. Furthermore, this study may establish a potential intervention threshold for future prospective clinical research examining the impact of FXIII supplementation therapy on the prognosis of patients with sICH.

## Supplementary Information

Below is the link to the electronic supplementary material.

Supplementary file1 (DOCX 30 KB)

Supplementary file2 (DOCX 29 KB)

## Data Availability

The datasets used or analyzed during the current study are available from the corresponding author on reasonable request.

## Abbreviations

sICH: Spontaneous intracerebral hemorrhage
HE: Hematoma expansion
SBP: Systolic blood pressure
MAP: Mean arterial pressure
GCS: Glasgow Coma Scale
OICH: Original intracerebral hemorrhage scale
mRS: Modified Rankin Scale
RBC: Red blood cell
WBC: White blood cell
INR: International normalized ratio
PT: Prothrombin
APTT: Activated partial thromboplastin time
TEG: Thromboelastography
FIB: Fibrinogen
FXIII: Coagulation factor XIII
