# Supplementary material for: Impact of FXIII Levels on Hematoma Expansion and Outcomes in Patients with Spontaneous Intracranial Hemorrhage: A Prospective Observational Cohort Study of 132 Patients
Source: Neurocrit Care. 2026 Apr 3;45(2):581–92. doi: 10.1007/s12028-026-02513-3 (PMC13642390; doi:10.1007/s12028-026-02513-3)
Supplement: Supplementary file 1 [file 12028_2026_2513_MOESM1_ESM.docx]

**Supplementary Table 1. Baseline Characteristics of The Dead and Surviving Patients with Spontaneous Intracerebral Hemorrhage (sICH)**

|  | Total (n = 132) | Survived (n = 103) | Died (n = 29) | *P* Value*^a^* |
| --- | --- | --- | --- | --- |
|  |  |  |  |  |
| **Age,** **Median [IQR]** | 59 (51, 70.25) | 58 (51, 68) | 64 (56, 75) | 0.024 |
| **Gender, n (%)** |  |  |  | 1 |
| **Male** | 92 (69.7) | 72 (69.9) | 20 (68.97) |  |
| **Female** | 40 (30.3) | 31 (30.1) | 9 (31.03) |  |
| **Hypertension, n (%)** |  |  |  | 0.023 |
| **No** | 39 (29.55) | 25 (24.27) | 14 (48.28) |  |
| **Yes** | 93 (70.45) | 78 (75.73) | 15 (51.72) |  |
| **SBP, Mean ± SD** | 165.9 ± 26.63 | 167.02 ± 24.16 | 161.93 ± 34.2 | 0.365 |
| **MAP, Mean ± SD** | 120.43 ± 19.41 | 121.33 ± 18.62 | 117.24 ± 22.03 | 0.318 |
| **GCS at admission, Median [IQR]** | 11 (8, 13) | 11 (9, 13) | 7 (4, 10) | < 0.001 |
| **OICH, Median [IQR]** | 2 (1, 3) | 1 (1, 2) | 3 (2, 4) | < 0.001 |
| **Na(mmol/L), Median [IQR]** | 139.3 (137.3, 142) | 139.8 (137.6, 142) | 138.1 (135.9, 141) | 0.286 |
| **Glucose(mmol/L), Median [IQR]** | 7.95 (6.8, 9.77) | 7.6 (6.55, 9.1) | 9.3 (8, 12.1) | < 0.001 |
| **RBC(×10^12^/L), Median [IQR]** | 4.54 (4.02, 4.96) | 4.72 (4.12, 5.07) | 4.19 (3.77, 4.75) | 0.002 |
| **WBC(×10^9^/L), Median [IQR]** | 10.28 (8, 12.94) | 10.22 (7.98, 12.53) | 10.73 (8.33, 15.7) | 0.343 |
| **Creatinine(µmol/L), Median [IQR]** | 66.35 (54.98, 79.78) | 66 (55.4, 79.05) | 70 (54, 81) | 0.813 |
| **INR, Median [IQR]** | 0.99 (0.95, 1.04) | 0.98 (0.95, 1.02) | 1 (0.96, 1.07) | 0.123 |
| **PT(s), Median [IQR]** | 12.8 (11.9, 13.22) | 12.7 (11.85, 13.2) | 13.1 (12.2, 13.5) | 0.08 |
| **APTT(s), Median [IQR]** | 31.15 (26.87, 33.9) | 31.2 (26.6, 33.9) | 30.6 (27.9, 33.1) | 0.961 |
| **FIB(g/L), Median [IQR]** | 3.04 (2.54, 3.52) | 3.03 (2.54, 3.46) | 3.08 (2.54, 3.91) | 0.686 |
| **D-dimer(mg/L), Median [IQR]** | 0.56 (0.35, 1.09) | 0.48 (0.32, 0.88) | 0.83 (0.5, 2.02) | 0.004 |
| **Platelet(×10^9^/L), Median [IQR]** | 197 (160, 227.25) | 195 (160, 227.5) | 198 (162, 219) | 0.627 |
| **Hemoglobin(g/L), Median [IQR]** | 138 (123.75, 148) | 140 (127, 150) | 132 (115, 140) | 0.009 |
| **Hematoma Expansion, n (%)** |  |  |  | 0.07 |
| **No** | 93 (70.45) | 77 (74.76) | 16 (55.17) |  |
| **Yes** | 39 (29.55) | 26 (25.24) | 13 (44.83) |  |
| **Baseline Volume(ml), Median [IQR]** | 15.89 (8, 39.5) | 14.22 (8, 31.5) | 49 (13.54, 74) | < 0.001 |
| **Location, n (%)** |  |  |  | 0.773 |
| **Deep gray matter** | 88 (66.67) | 70 (67.96) | 18 (62.07) |  |
| **Infratentorial** | 17 (12.88) | 13 (12.62) | 4 (13.79) |  |
| **Lobar** | 27 (20.45) | 20 (19.42) | 7 (24.14) |  |
| **Presence of IVH, n (%)** |  |  |  | 0.01 |
| **No** | 71 (53.79) | 62 (60.19) | 9 (31.03) |  |
| **Yes** | 61 (46.21) | 41 (39.81) | 20 (68.97) |  |
| **Spot Sign, n (%)** |  |  |  | 0.674 |
| **No** | 66 (50) | 53 (51.46) | 13 (44.83) |  |
| **Yes** | 66 (50) | 50 (48.54) | 16 (55.17) |  |
| **Surgical, n (%)** |  |  |  | 1 |
| **No** | 63 (47.73) | 49 (47.57) | 14 (48.28) |  |
| **Yes** | 69 (52.27) | 54 (52.43) | 15 (51.72) |  |
| **FXIII (mg/L), Median [IQR]** | 9.81 (7.88, 18.1) | 11.68 (8.54, 19.15) | 7.13 (5.95, 8.34) | < 0.001 |
| **FXIII (mg/L), n (%)** |  |  |  | < 0.001 |
| **>10mg/L** | 63 (47.73) | 61 (59.22) | 2 (6.9) |  |
| **≤10mg/L** | 69 (52.27) | 42 (40.78) | 27 (93.1) |  |

Abbreviations: sICH, spontaneous intracerebral hemorrhage; SBP, systolic blood pressure; MAP, mean arterial pressure; GCS, Glasgow Coma Scale; OICH, original intracerebral hemorrhage scale; RBC, red blood cell; WBC, white blood cell; INR, international normalized ratio; PT, prothrombin; APTT, activated partial thromboplastin time; FIB, fibrinogen; FXIII, coagulation factor XIII.

*^a^* *P* values not adjusted for multiple comparisons. Statistical significance determined by chi-square test, Student’s t-test or Mann-Whitney tests.
