# Supplementary material for: Impact of FXIII Levels on Hematoma Expansion and Outcomes in Patients with Spontaneous Intracranial Hemorrhage: A Prospective Observational Cohort Study of 132 Patients
Source: Neurocrit Care. 2026 Apr 3;45(2):581–92. doi: 10.1007/s12028-026-02513-3 (PMC13642390; doi:10.1007/s12028-026-02513-3)
Supplement: Supplementary file 2 [file 12028_2026_2513_MOESM2_ESM.docx]

**Supplementary Table 2. Baseline Characteristics and Functional Outcomes of Patients with Spontaneous Intracerebral Hemorrhage (sICH)**

|  | Total (n = 132) | mRS 0-2 (n = 35) | mRS 3-6 (n = 97) | *P* Value*^a^* |
| --- | --- | --- | --- | --- |
|  |  |  |  |  |
| **Age, Median [IQR]** | 59 (51, 70.25) | 57 (48.5, 65) | 60 (52, 73) | 0.065 |
| **Gender, n (%)** |  |  |  | 0.964 |
| **Male** | 92 (69.7) | 25 (71.43) | 67 (69.07) |  |
| **Female** | 40 (30.3) | 10 (28.57) | 30 (30.93) |  |
| **Hypertension, n (%)** |  |  |  | 0.036 |
| **No** | 39 (29.55) | 5 (14.29) | 34 (35.05) |  |
| **Yes** | 93 (70.45) | 30 (85.71) | 63 (64.95) |  |
| **SBP, Mean ± SD** | 165.9 ± 26.63 | 163.29 ± 24.38 | 166.85 ± 27.45 | 0.5 |
| **MAP, Mean ± SD** | 120.43 ± 19.41 | 120.71 ± 18.86 | 120.33 ± 19.69 | 0.921 |
| **GCS at admission, Median [IQR]** | 11 (8, 13) | 14 (12.5, 14.5) | 10 (7, 12) | < 0.001 |
| **OICH, Median [IQR]** | 2 (1, 3) | 1 (1, 2) | 2 (1, 3) | < 0.001 |
| **Na(mmol/L), Median [IQR]** | 139.3 (137.3, 142) | 141 (137.75, 142.8) | 138.3 (137.2, 141.4) | 0.051 |
| **Glucose(mmol/L), Median [IQR]** | 7.95 (6.8, 9.77) | 7.8 (6.65, 9.5) | 8 (6.9, 10.3) | 0.455 |
| **RBC(×10^12^/L), Median [IQR]** | 4.54 (4.02, 4.96) | 4.82 (4.34, 5.15) | 4.39 (3.96, 4.89) | 0.028 |
| **WBC(×10^9^/L), Median [IQR]** | 10.28 (8, 12.94) | 9.8 (8.16, 11.11) | 10.51 (7.68, 13.28) | 0.504 |
| **Creatinine(µmol/L), Median [IQR]** | 66.35 (54.98, 79.78) | 61 (54.5, 71) | 69 (55, 81.8) | 0.149 |
| **INR, Median [IQR]** | 0.99 (0.95, 1.04) | 0.96 (0.94, 1) | 1 (0.95, 1.06) | 0.02 |
| **PT(s), Median [IQR]** | 12.8 (11.9, 13.22) | 12.5 (11.1, 12.95) | 12.9 (12.3, 13.4) | 0.006 |
| **APTT(s), Median [IQR]** | 31.15 (26.87, 33.9) | 30.9 (24.5, 33.4) | 31.2 (27.9, 34) | 0.207 |
| **FIB(g/L), Median [IQR]** | 3.04 (2.54, 3.52) | 3 (2.56, 3.54) | 3.08 (2.51, 3.47) | 0.922 |
| **D-dimer(mg/L), Median [IQR]** | 0.56 (0.35, 1.09) | 0.41 (0.28, 0.79) | 0.59 (0.38, 1.42) | 0.004 |
| **Platelet(×10^9^/L), Median [IQR]** | 197 (160, 227.25) | 195 (177, 227.5) | 198 (145, 225) | 0.394 |
| **Hemoglobin(g/L), Median [IQR]** | 138 (123.75, 148) | 142 (131, 152.5) | 138 (123, 147) | 0.076 |
| **Hematoma Expansion, n (%)** |  |  |  | 0.012 |
| **No** | 93 (70.45) | 31 (88.57) | 62 (63.92) |  |
| **Yes** | 39 (29.55) | 4 (11.43) | 35 (36.08) |  |
| **Baseline Volume(ml), Median [IQR]** | 15.89 (8, 39.5) | 8 (5, 15.51) | 22 (10.32, 48) | < 0.001 |
| **Location, n (%)** |  |  |  | 0.151 |
| **Deep gray matter** | 88 (66.67) | 19 (54.29) | 69 (71.13) |  |
| **Infratentorial** | 17 (12.88) | 6 (17.14) | 11 (11.34) |  |
| **Lobar** | 27 (20.45) | 10 (29.59) | 17 (17.53) |  |
| **Presence of IVH, n (%)** |  |  |  | 0.508 |
| **No** | 71 (53.79) | 21 (60) | 50 (51.55) |  |
| **Yes** | 61 (46.21) | 14 (40) | 47 (48.45) |  |
| **Spot Sign, n (%)** |  |  |  | 0.237 |
| **No** | 66 (50) | 14 (40) | 52 (53.61) |  |
| **Yes** | 66 (50) | 21 (60) | 45 (46.39) |  |
| **Surgical, n (%)** |  |  |  | 0.27 |
| **No** | 63 (47.73) | 20 (57.14) | 43 (44.33) |  |
| **Yes** | 69 (52.27) | 15 (42.86) | 54 (55.67) |  |
| **FXIII (mg/L), Median [IQR]** | 9.81 (7.88, 18.1) | 22.18 (18.08, 26.69) | 9 (7.13, 11.09) | < 0.001 |
| **FXIII (mg/L), n (%)** |  |  |  | < 0.001 |
| **>10mg/L** | 63 (47.73) | 34 (97.14) | 29 (29.9) |  |
| **≤10mg/L** | 69 (52.27) | 1 (2.86) | 68 (70.1) |  |

Abbreviations: sICH, spontaneous intracerebral hemorrhage; SBP, systolic blood pressure; MAP, mean arterial pressure; GCS, Glasgow Coma Scale; OICH, original intracerebral hemorrhage scale; RBC, red blood cell; WBC, white blood cell; INR, international normalized ratio; PT, prothrombin; APTT, activated partial thromboplastin time; FIB, fibrinogen; FXIII, coagulation factor XIII.

*^a^* *P* values not adjusted for multiple comparisons. Statistical significance determined by chi-square test, Student’s t-test or Mann-Whitney tests.
